# Time-series analysis for porcine reproductive and respiratory syndrome in the United States

**DOI:** 10.1371/journal.pone.0195282

**Published:** 2018-04-03

**Authors:** Andréia Gonçalves Arruda, Carles Vilalta, Pere Puig, Andres Perez, Anna Alba

**Affiliations:** 1 Department of Veterinary Preventive Medicine, The Ohio State University, Columbus, Ohio, United States of America; 2 Department of Veterinary Population Medicine, University of Minnesota, St. Paul, Minnesota, United States of America; 3 Department of Mathematics, Autonomous University of Barcelona, Barcelona, Spain; Sun Yat-Sen University, CHINA

## Abstract

Industry-driven voluntary disease control programs for swine diseases emerged in North America in the early 2000’s, and, since then, those programs have been used for monitoring diseases of economic importance to swine producers. One example of such initiatives is Dr. Morrison’s Swine Health Monitoring Project, a nation-wide monitoring program for swine diseases including the porcine reproductive and respiratory syndrome (PRRS). PRRS has been extensively reported as a seasonal disease in the U.S., with predictable peaks that start in fall and are extended through the winter season. However, formal time series analysis stratified by geographic region has never been conducted for this important disease across the U.S. The main objective of this study was to use approximately seven years of PRRS incidence data in breeding swine herds to conduct time-series analysis in order to describe the temporal patterns of PRRS outbreaks at the farm level for five major swine-producing states across the U.S. including the states of Minnesota, Iowa, North Carolina, Nebraska and Illinois. Data was aggregated retrospectively at the week level for the number of herds containing animals actively shedding PRRS virus. Basic descriptive statistics were conducted followed by autoregressive integrated moving average (ARIMA) modelling, conducted separately for each of the above-mentioned states. Results showed that there was a difference in the nature of PRRS seasonality among states. Of note, when comparing states, the typical seasonal pattern previously described for PRRS could only be detected for farms located in the states of Minnesota, North Carolina and Nebraska. For the other two states, seasonal peaks every six months were detected within a year. In conclusion, we showed that epidemic patterns are not homogeneous across the U.S, with major peaks of disease occurring through the year. These findings highlight the importance of coordinating alternative control strategies in different regions considering the prevailing epidemiological patterns.

## Introduction

Industry-driven voluntary swine disease control programs emerged in North America in the early 2000’s. Since then, those programs have been widely used for monitoring and surveillance of diseases of economic importance to swine producers. Even though these control programs have been relatively common for PRRS in North America [1–3], data emerging from those programs have rarely been assessed for research purposes.

One example of such initiatives is the Dr. Morrison’s Swine Health Monitoring Project (**MSHMP**), a nation-wide monitoring program for swine diseases that affect and are economically important for the U.S. swine industry, including the porcine reproductive and respiratory syndrome (**PRRS**), the porcine epidemic diarrhea (**PED**), and Senecavirus A. The University of Minnesota led this initiative starting in 2011, in collaboration with the American Association of Swine Veterinarians (**AASV**), the National Pork Board, and the Swine Health Information Center. Since then, the MSHMP has served as a means to capture the occurrence of infectious diseases for situations in which there is an absence of a regulatory framework [4]. At the time of writing of this manuscript, in December 2017, the program represented a considerable portion of breeding herds in the country (approximately 45%; [5]) and collected disease incidence data on a weekly basis.

Porcine reproductive and respiratory syndrome has been previously reported as a seasonal disease with predictable peaks starting during mid-October [6]; therefore, it is not uncommon that farm-level prevention efforts are intensified during this time. However, previous research conducted by our research group suggested that PRRS virus transmissibility as captured by the time-dependent reproduction number is incredibly region- and system-dependent [7], for reasons that could potentially include (but are not limited to) demographic and environmental factors [8, 9]. Even though the swine industry commonly reports PRRS as a seasonal disease, systematic disaggregation of data into regions and systems combined with formal and full assessment and comparison of seasonality PRRS patterns across different US regions has never been published in the peer-reviewed literature. One of the reasons for the lack of such information is that consistent collection of longitudinal disease data over time has been traditionally uncommon for the swine industry in North America. However, after years of data collection, the MSHMP database offered a unique opportunity for conducting, for the first time, temporal analysis such as the one described here.

Time-series analysis is a field in development, and, even though methods are currently available; these have not been largely explored for modelling of infectious diseases in food animal production systems. One of the few studies published in the peer-reviewed literature used cattle mortality data from over nine years from a region in Spain to apply autoregressive integrated moving average (**ARIMA**) modelling and hierarchical time series to determine basal patterns of bovine fallen stock at different scales (region, province, county and municipality) [10]. The researchers concluded that the time series approaches were useful as tools for comparing mortality patterns across different populations over time [10].

The main objective of this study was to use time-series analysis to describe the temporal patterns of PRRS at the farm level for five major swine-producing states across the U.S. Specifically, our aim was to investigate whether yearly patterns commonly described for PRRS were in fact conserved across different U.S. states. Our main hypothesis was that there was one yearly seasonal peak would be found for PRRS for all examined states and systems (winter season). Evaluating that hypothesis will help to understand the epidemiological dynamics of PRRS in the U.S. Ultimately, that information will help to design and implement surveillance and control programs for one of the most financially devastating diseases of swine in the country.

## Materials and methods

### Farm selection and outcome definition

The source population for this project corresponded to breeding herds (herds that contain sows) that participated in the Dr. Morrison’s Swine Health Monitoring Project from July 2009 (starting on the 26^th^ week of 2009) to October 2016 (finishing on the 41^st^ week of 2016). Even though the MSHMP source population includes over 1,000 participating sow farms, only swine herds that contributed with complete data from those previously mentioned years were enrolled in this study, so that a closed population could be examined.

Data were aggregated retrospectively at the week level for the number of herds that were considered as having an active outbreak each week. Farms would be allocated to that category as soon as outbreaks were identified and reported by herd veterinarians and based on a number of features. Those features included one or a combination of the conditions of presence of PRRS-consistent clinical signs and identification of the virus via diagnostic testing, and/or isolation of a different PRRS virus strain as compared to previous existing strains in the herd; as described elsewhere [7]. Once herds reported an outbreak, they were considered ‘status 1’ or ‘positive unstable’ following AASV PRRS classification guidelines [11] until there was absence of clinical signs and no detectable viremia in weaned piglets for a minimum of 90 days (tested at least every 30 days) [11].

### Exploratory descriptive analysis

Descriptive analysis was conducted using R v.3.2.3 (R Core Team, 2013). First, we characterized the number of sites per region, the mean (standard deviation [**SD**]) number of animals in the herds and the mean number of farms containing animals actively shedding PRRS virus (considering a closed population) over time. Second, descriptive analysis of the time series was conducted by plotting the raw counts of sites classified as PRRS ‘status 1’ for all the source population and for the subpopulations according to geographical region. Statistics such as mean, median, minimum, maximum, variance and autocovariance functions were analyzed for each time series separately to determine basic patterns in the data and to aid in the decision of whether an alternative model for low counts [12] would be needed during time series analysis.

### Time series analysis

The level of resolution used in this study was weeks because this was the finer level of resolution available from the MSHMP and avoided possible weekend or holiday effects. Counts of swine farms containing animals actively shedding PRRS virus was used as the main outcome of interest. Data from a total of 388 weeks were available for analysis (2009–2016). Data were stratified at the state level and separately analyzed. The five states examined included Minnesota (MN), Iowa (IA), North Carolina (NC), Nebraska (NE), and Illinois (IL).

Autoregressive integrated moving average (ARIMA) modelling was conducted as described elsewhere [10]. In brief, the following steps were taken for construction of models: first, data were decomposed and plotted to visually assess the presence and characteristic of the trend and the seasonality, as well as the autocorrelation (**ACF**) and the partial autocorrelation (**PACF**) plots. Second, autoregressive moving average modelling was performed, including trend and seasonality terms as covariates. Here, the seasonality was represented by including trigonometric covariates (or Fourier terms). Seasonal cycles of one year and six, four and three months were tested by multivariate linear regression using the least square method [10]. The selection of the most appropriate model for each time series of the study was based on three criteria: assessment of the Akaike Information Criterion (**AIC**), statistical significance of the parameters of the model at a reasonable significance level of 0.05, the Box-Pierce test, and the evaluation of the behaviour of the residuals checking its autocorrelation and partial autocorrelation function. For series in which the counts contained a considerable number of zeros, a generalized linear autoregressive moving average model with a Poisson structure was also attempted (‘glarma’ R package; [13]), with model selection again being prioritized as described above.

## Results

**Fig 1:**
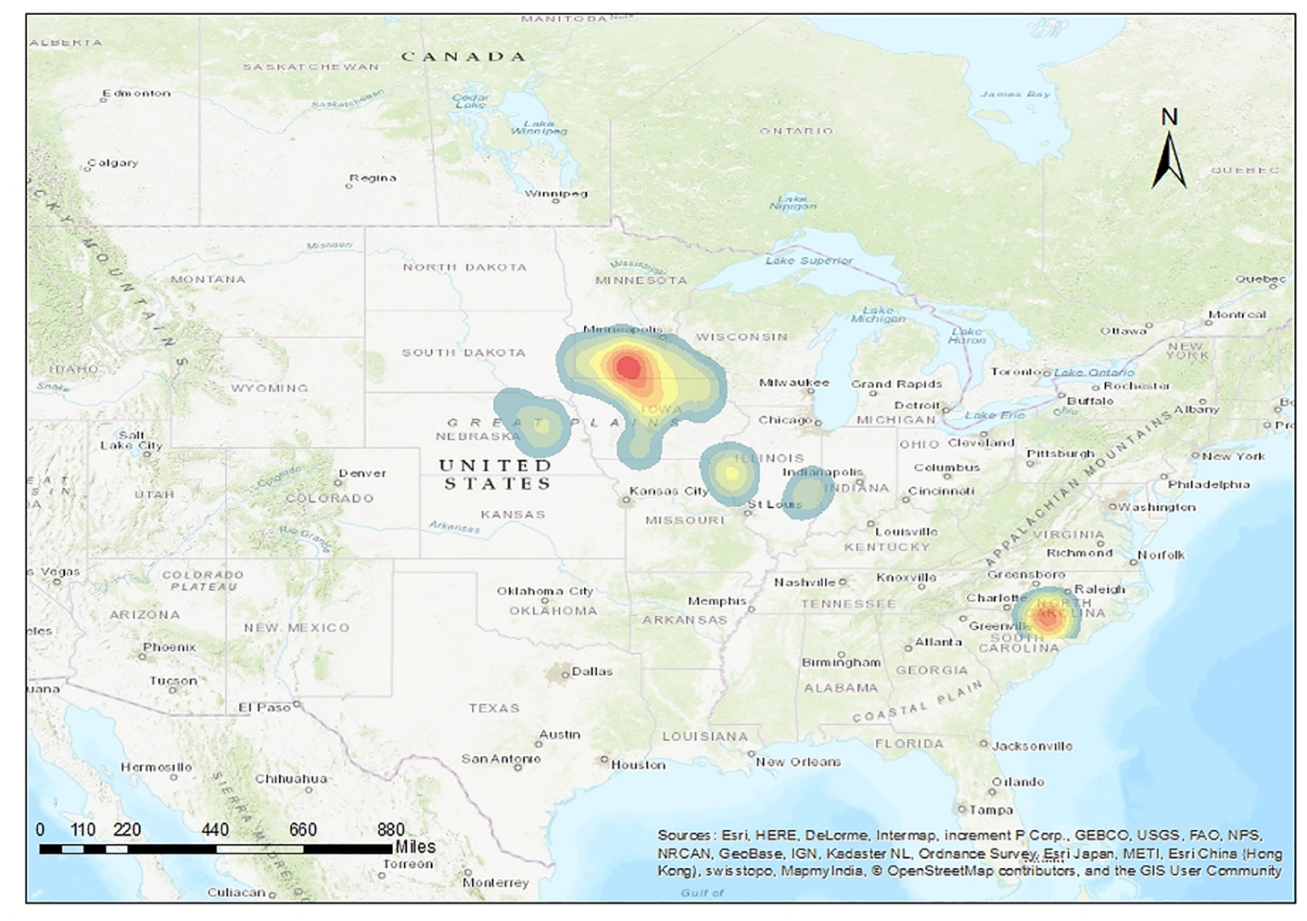
Kernel smoothed map created using ArcMap 10.3 showing geographical distribution of 268 breeding swine sites that contributed with complete data for this project. Sites were participants of the Dr Morrison’s Swine Health Monitoring Project during 2009–2016.

There were 268 farms included in this study; the majority of participating farms were located in the swine dense regions of Minnesota and Iowa. Fig 1 shows the distribution of MSHMP participant farms within the five states examined herein.

Basic farm descriptors including average number of farms, average number of animals per farm and mean prevalence of PRRS virus positive farms by state are shown on Table 1.

**Table 1:** Description of swine sites participating in this study including mean number of animals per site and mean percent of PRRS virus positive farms, stratified by region and system.

Region or System	Number of sites	Mean number of animals (SD) per site	Mean percent of PRRS positive farms^1^ (median, min, max)
Minnesota	81	2666.02 (1120.96)	26.16 (26.00, 5.00, 60.00)
Iowa	72	3543.71 (1218.69)	31.68 (32.00, 10.00, 56.00)
North Carolina	45	2342.222 (974.09)	7.87 (7.00, 0.00, 27.00)
Nebraska	30	4041.60 (2514.63)	15.27 (17.00, 0.00, 40.00)
Illinois	40	4018 (2103.74)	14.06 (15.00, 0.00, 28.00)

^1^Positive farms were defined as ‘status 1’ farms according to AASV guidelines (PRRS virus positive farms; Holtkamp et al., 2011)

**Fig 2:**
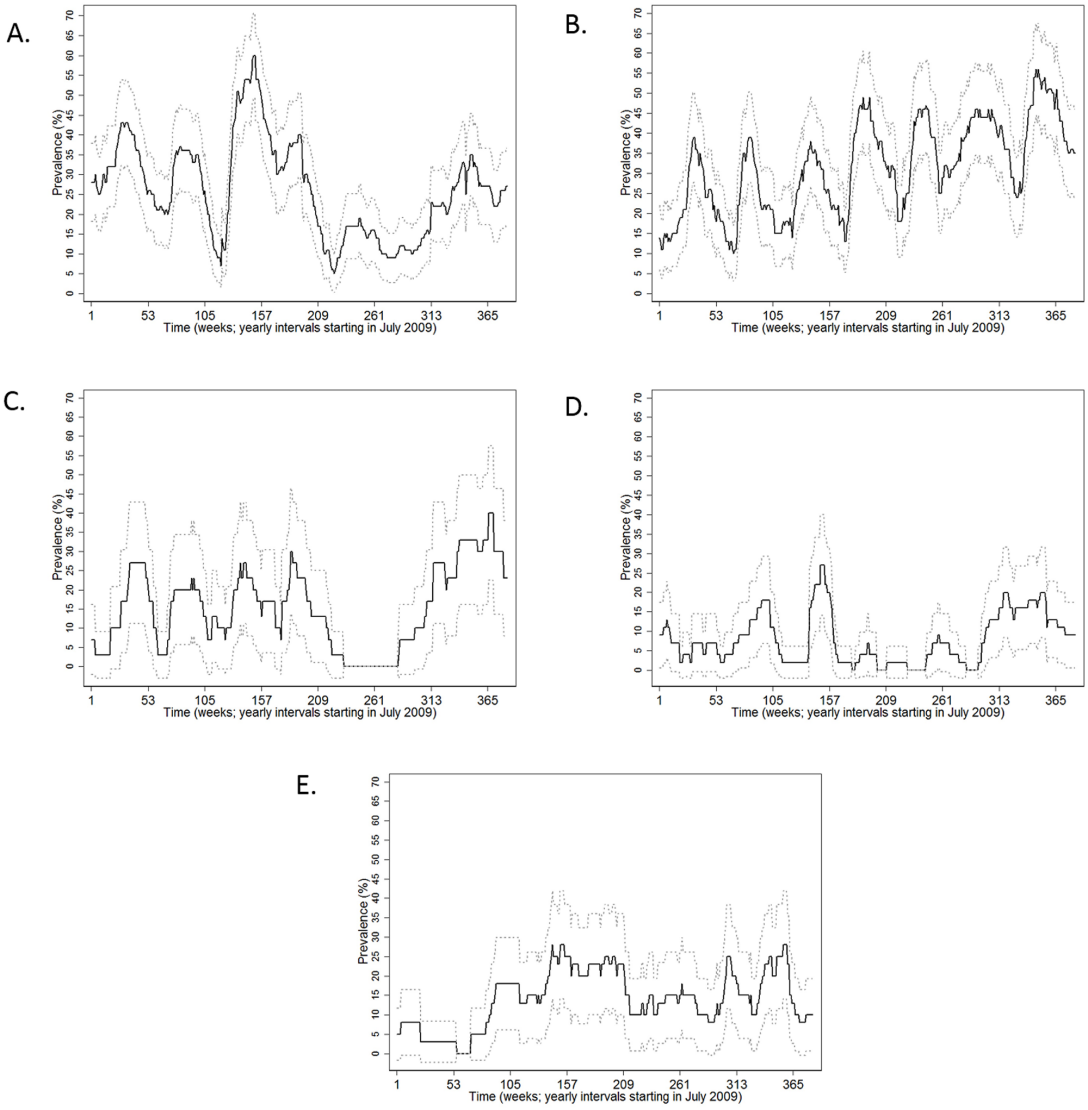
Graphs showing the prevalence (black line) and upper and lower 95% confidence intervals (grey dotted lines) of PRRS virus positive farms for the five different U.S. states participating in this study: A: Minnesota; B: Iowa; C: Nebraska, D: North Carolina and E: IllinoisThe best ARIMA models according to our selection criteria are provided on Table 2.

Fig 2 depicts the prevalence, upper and lower 95% confidence intervals of farms containing animals actively shedding PRRS virus over the weeks examined in this study.

**Fig 3:**
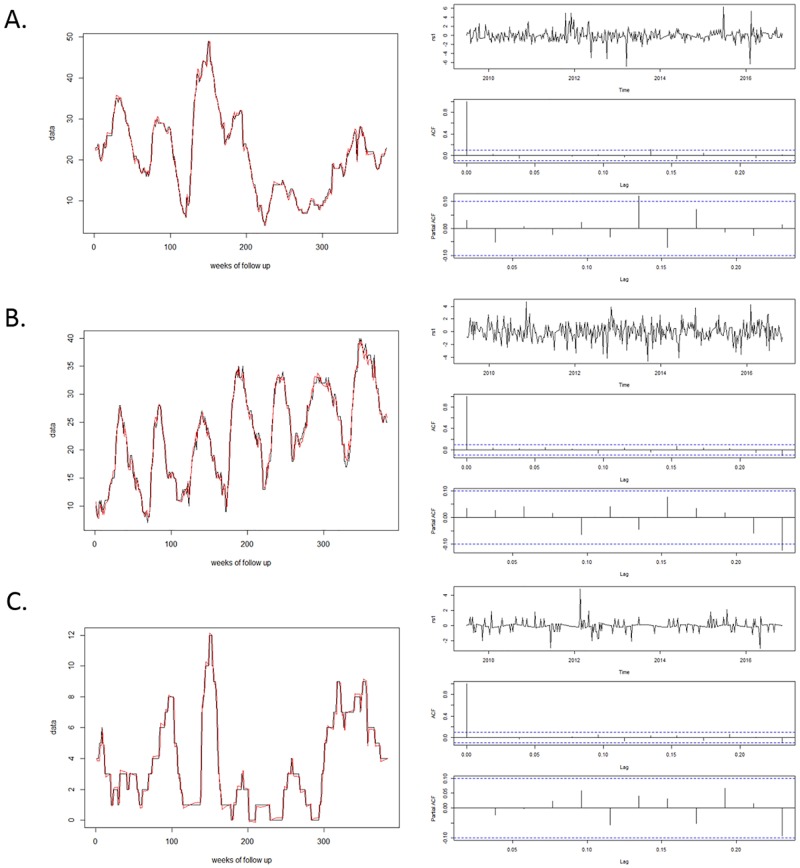
Model fit (left graph) and diagnostics (right graph) for time series models from: A. Minnesota; B. Iowa; and C. North Carolina. Diagnostics include plotting of standardized residuals (top right graph), autocorrelation function (ACF, middle right graph), and partial autocorrelation function (PACF, bottom right graph).

**Fig 4:**
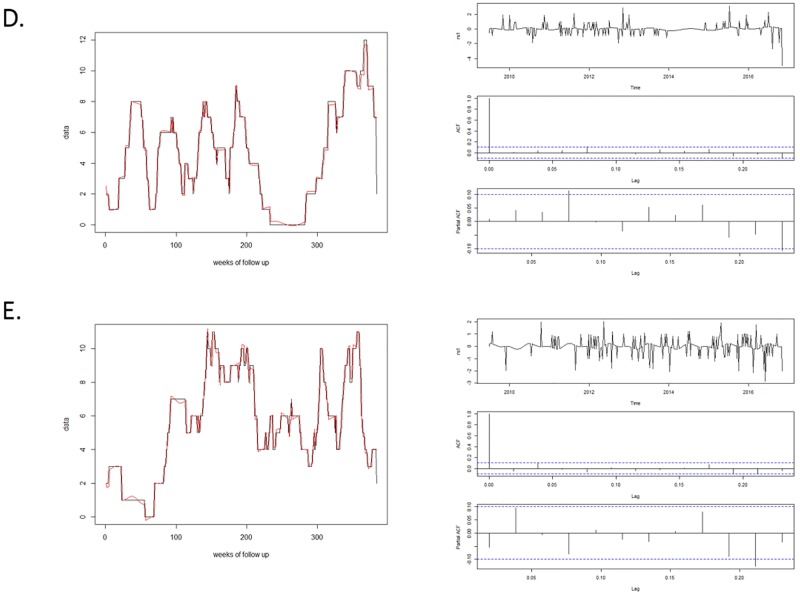
Model fit (left graph) and diagnostics (right graph) for time series models from: D. Nebraska; and E. Illinois. Diagnostics include plotting of standardized residuals (top right graph), autocorrelation function (ACF, middle right graph), and partial autocorrelation function (PACF, bottom right graph).

There was a difference in the nature of PRRS seasonality among states. A fall/ winter seasonal pattern was only detected for farms located in the states of Minnesota, North Carolina and Nebraska. For the other two states (Iowa and Illinois), two seasonal peaks were detected within a year: one every 6 months (biannual). Figs 3 and 4 shows the fit and diagnostics for all final models, and covariate estimates, standard errors and AIC values are provided on Table 2. All models appeared to fit well considering the graphs and all p-values from the Box-Pierce test were p > 0.05.

**Table 2:** Final autoregressive integrated moving average (ARIMA) models including trend and seasonality as covariates.

U.S. state	ARIMA type	Final Models^1^							
Minnesota	(1,1,1)	X_t_−X_t-1_ =	– 0.61 cos (2πt/52)	– 5.60 sin (2πt/52)	+ Y_t_				
		s.e.	1.07	1.08					
		Y_t_ =	0.85Y_t-1_	+ Z_t_	– 0.75Z_t-1_				
		s.e.	0.09		0.11				
		AIC =	1263.94						
Iowa	(1,0,0)	X_t_ =	13.43	+ 0.05t	– 1.94 cos (2πt/52)	– 6.75 sin (2πt/52)	+ 0.65 cos (2πt/26)	+ 1.95 sin (2πt/26)	+ Y_t_
		s.e.		0.006	0.61	0.62	0.37	0.37	
		Y_t_ =	0.90Y_t-1_						
		s.e.	0.02						
		AIC =	1296.44						
North Carolina	(1,1,1)	X_t_−X_t-1_ =	– 1.27 cos (2πt/52)	– 1.01 sin (2πt/52)	+ Y_t_				
		s.e.	0.43	0.43					
		Y_t_ =	0.78 Y_t-1_	+ Z_t_	– 0.72 Z_t_				
		s.e.	0.15		0.17				
		AIC =	698.72						
Nebraska	(1,0,0)	X_t_ =	4.20	+ 0.38 cos (2πt/52)	– 1.10 sin (2πt/52)	+ Y_t_			
		s.e.	1.28	0.36	0.37				
		Y_t_ =	0.98 Y_t-1_						
		s.e.	0.01						
		AIC =	728.55						
Illinois	(0,1,0)	X_t_−X_t-1_ =	0.90 cos (2πt/52)	– 0.91 sin (2πt/52)	+ 0.01 cos (2πt/26)	- 0.46 sin (2πt/26)			
		s.e.	0.34	0.34	0.17	0.17			
		AIC =	674.42						

^1^Contains coefficients, SE (standard errors for the respective coefficients) and AIC (Akaike information criterion); p-value < 0.05 was deemed as statistically significant

## Discussion

Autoregressive integrated moving average (ARIMA) modelling was used here to describe seasonal patterns of PRRS outbreaks in swine farms across five states of the United States. This stratification allowed for the comparison across different subpopulations of farms and identification of important differences in disease prevalence over time, which, prior to the study here, were believed to be homogeneous in the country. The main finding of this study was that PRRS seasonality varies according to geographical region, and the commonly referred “PRRS season” is not necessarily the only time of increase in disease incidence. Even though PRRS is anecdotally considered a seasonal disease, we showed that, for some regions among the U.S., there are other considerable peaks of disease during the year.

Sporadic cases have anecdotally been referred to as “summer outbreaks”. Even though it has been reported that cold temperatures (winter season) and low relative humidity favor PRRS virus survival [14], some factors might help explain these off-winter incidence of PRRS cases. First, during summer months there is larger commingling of animal producers in state fairs or animal shows [15], which could facilitate disease transmission in the absence of adequate biosecurity practices [16, 17]. Second, lack of awareness of occurrence of other PRRS cases outside the neighborhood and system could contribute to a relaxed attitude towards some biosecurity measures during these months (e.g. less truck washing, drying and disinfecting events), allowing for virus spread [18]. Lastly, considering the volume of activities that occur in the current structure of the swine industry [19], it is not surprising that there are a lot of opportunities for the pathogens to spread via non-obvious manner such as through feed trucks, fomite and personnel, therefore; even though the virus might be easily transmitted during winter months, the amount of activity is enough for these sporadic events to occur in considerable amounts.

Another interesting finding from this study was the presence of an alternating trend for all examined states within of the U.S., except for the state of Iowa, the largest pork producing states in the country (approximately 31.4% of the total US hog and pig inventory, [20]), which had an increasing linear trend over the examined years. The period during which this changing point occurred was between May 2013 and late March 2014, which, not surprisingly, coincides with the calendar year in which the U.S. had a porcine epidemic diarrhea outbreak [21, 22]. It has been previously reported that during 2013–2014, there were significantly fewer PRRS cases for reasons that are still hypothesized [22]. Due to the fact that an unpredictable event occurred in the population examined here, our data were not considered robust enough for division into testing and training and potential prediction. Such analysis may be, however, conducted in the future, when enough data would be gathered over the years.

Strengths of the study here are the large sample size, the geographic representation of the major pig-producing U.S. states, and the potential impact of the research, given that this is the disease that cause the most far-reaching impact to the swine industry of the country. Furthermore, data available included over nine years of weekly disease data classified in a standardized manner, which is somewhat rare for an industry-based swine project. ARIMA models were used because they are powerful tools that can be directly fitted using standard packages in R. However the use of families of continuous-time ARMA processes [23], or Integer-valued AR processes [12] could be also worth exploring in further research.

An important limitation of the study is that solely breeding herds were included as these were the only production types reporting disease status to the MSHMP; therefore, extrapolation of findings to growing pig populations should be made with caution. However, the most valuable animals in the value chain of the industry were included in the assessment, supporting the importance and impact of the findings. We also acknowledge misclassification bias is possible for the cases in which farms were not correctly classified by the veterinarian as positive shedding. This would be especially a problem for herds that have some kind of underlying immunity (e.g. vaccinated or live-inoculated herds). We realize that, the utilized scheme of classification does not fully detect farms that have low prevalence of animals that are shedding the virus. However, this was the best available method for classifying swine herds in regards to PRRS at the present time, and we the advantage relies on the fact that a standardized method was used throughout the study period, therefore; if this misclassification occurred, it would be consistent across farms, which we believe would dramatically change our conclusions.

Finally, emergence of PED at a given point during the study period prevented researchers to develop predictive models at this instance, but the current work will serve as basis for establishing a disease baseline by region, as well as to bring awareness that PRRS is a seasonal disease, and that there may be more than one peak in PRRS cases during the course of an year. Farm and region-level variables were not available to us, but could be collected to investigate potential drivers of the state and system-level differences reported herein.

In conclusion, we showed that PRRS seasonal patterns are not homogeneous across the U.S., with some important pork producing states having biannual PRRS peaks instead of the previously reported winter peak. Findings from this study highlight the importance of coordinating alternative control strategies in different regions considering the prevailing epidemiological patterns, and the need to reinforce strict biosecurity practices beyond the typically described “PRRS season”.
